# Structural Characterization of a Gcn5-Related N-Acetyltransferase from *Staphylococcus aureus*

**DOI:** 10.1371/journal.pone.0102348

**Published:** 2014-08-13

**Authors:** Parul Srivastava, Yogesh B. Khandokar, Crystall M. D. Swarbrick, Noelia Roman, Zainab Himiari, Subir Sarker, Shane R. Raidal, Jade K. Forwood

**Affiliations:** 1 School of Biomedical Sciences, Charles Sturt University, Wagga Wagga, New South Wales, Australia; 2 School of Animal and Veterinary Sciences, Charles Sturt University, Wagga Wagga, New South Wales, Australia; University Paris Diderot-Paris 7, France

## Abstract

The Gcn5-related N-acetyltransferases (GNATs) are ubiquitously expressed in nature and perform a diverse range of cellular functions through the acetylation of small molecules and protein substrates. Using activated acetyl coenzyme A as a common acetyl donor, GNATs catalyse the transfer of an acetyl group to acceptor molecules including aminoglycoside antibiotics, glucosamine-6-phosphate, histones, serotonin and spermidine. There is often only very limited sequence conservation between members of the GNAT superfamily, in part, reflecting their capacity to bind a diverse array of substrates. In contrast, the secondary and tertiary structures are highly conserved, but then at the quaternary level there is further diversity, with GNATs shown to exist in monomeric, dimeric, or tetrameric states. Here we describe the X-ray crystallographic structure of a GNAT enzyme from *Staphyloccocus aureus* with only low sequence identity to previously solved GNAT proteins. It contains many of the classical GNAT motifs, but lacks other hallmarks of the GNAT fold including the classic β-bulge splayed at the β-sheet interface. The protein is likely to be a dimer in solution based on analysis of the asymmetric unit within the crystal structure, homology with related GNAT family members, and size exclusion chromatography. The study provides the first high resolution structure of this enzyme, providing a strong platform for substrate and cofactor modelling, and structural/functional comparisons within this diverse enzyme superfamily.

## Introduction

The Gcn5-related N-acetyltransferases (GNATs) are a very large enzyme superfamily with more than 10,000 members identified across all kingdoms of life [1]. They were first identified as aminoglycoside acetyltransferases from bacteria that developed antibiotic resistance to kanamycin and gentamicin [1]. The GNATs catalyse the transfer of an acetyl group from acetyl CoA to the primary amine of substrates including antibiotics aminoglycosides, glucosamine-6-phosphate, histones, serotonin, spermine, spermidine, and other small molecules [2]–[6]. In spite of the substrate and functional diversity, the basic structure of GNAT members is highly conserved. Members of the family share a common fold known as the GNAT fold, comprised of 6–7 anti-parallel β-strands and 4 α-helices in the topology β1-α1-α2-β2-β3-β4-α3-β5-α4-β6-β7.

The GNAT fold contains four conserved motifs A–D, arranged in the order C, D, A and B in the primary sequence. The most highly conserved motif across the superfamily is motif A, followed by motif B, D, and C. The hallmark of motif A is the “P-loop”, connecting helix α3 and strand β4, which plays an essential role in binding the β-mercaptoethylamine and pantothenic acid moieties of acetyl-CoA. Motif B spans conserved regions within α-helix 4 and is involved in binding the 3′, 5′-adenosine diphosphate of acetyl-CoA. Motif D, encompassing β2 and β3 strands is not directly involved in substrate or cofactor binding, but stabilises core structural elements of the protein, while motif C, located at the N-terminus of the protein, is the least conserved with some histone N-acetyltransferases lacking this motif completely. These motifs together comprise the common structural core known as GNAT domain. A signature of the GNAT fold is a splay between β4 and β5 strands, forming a V-shape opening in the central β sheet which is crucial in the transfer of acetyl group and binding of acetyl-CoA [7].

Whilst cell regulation through acetylation has been well characterised in eukaryotes, the role of protein acetylation within prokaryotes has only emerged recently, providing support that acetylation based regulation is an important and universal process. *Staphylococcus aureus*, an important pathogenic and increasingly multi-drug resistant bacterium, contains 35 putative GNAT enzymes, many of which remain uncharacterised both functionally and structurally. It is also an opportunistic human pathogen and frequent cause of infection ranging from mild to life threatening illnesses including bacteremia, meningitis, osteomyelitis, liver cirrhosis, keratitis, pneumonia, septic phlebitis and endocarditis [8], [9]. Moreover, rates of *S. aureus* infections have increased over past decade as has antibiotic resistance to commonly used antibiotics including rifampicin, vancomycin and methicillin [10], [11]. Resistance towards the aminoglycoside antibiotics can occur through a range of mechanisms including aminoglycoside modifying enzymes, ribosomal mutations, or excretion of the aminoglycoside. Aminoglycoside-modifying enzymes can inactivate antibiotics by covalently attaching either a phosphate, nucleotide, or acetyl moiety to either the amine or the alcohol key functional group (or both groups) of the antibiotic, changing the charge or sterically hindering the antibiotic [12], [13]. Thus, characterisation of proteins capable of playing a role in antibiotic resistance and regulatory functions within important pathogenic bacteria provides an important platform for rational drug design, development of new inhibitors, and an enhanced understanding of the putative functional roles.

Here, we describe the structure of an uncharacterised, GNAT family member from *S. aureus*. Our structure confirms that the protein exhibits many of the classical GNAT motifs, has high structural similarity with the phosphinoacetyl GNAT proteins, and is likely to exist as a dimer in solution based on biophysical and crystallographic properties.

## Materials and Methods

### Cloning and expression

The gene encoding the putative N-acetyltransferase from *S. aureus* subsp. aureus Mu50 NP_373053 (SaGNAT) was PCR amplified from genomic DNA purchased from American Type Cell Culture (ATCC), and cloned into the expression vector pMCSG21. The fidelity of the clone was confirmed by DNA sequencing and the plasmid transformed into *E. coli* BL21 (DE3) pLysS for recombinant expression. A 5 ml Luria–Bertani (LB) broth starter culture containing 100 µg/ml spectinomycin was used to inoculate 500 ml of auto-induction media [14] containing 100 µg/ml spectinomycin grown at 25°C for 24 h. The cells were harvested by centrifugation and the cell pellet resuspended in 50 ml of His buffer A (50 mM Phosphate buffer pH 8.0, 300 mM NaCl, 20 mM Imidazole) and stored at −20°C.

### Protein purification and crystallisation

The *E. coli* cells were lysed by 2 repetitive freeze-thaw cycles in the presence of 20 mg of lysozyme, and the lysate centrifuged at 15,000 rpm for 30 min. The supernatant was filtered through a 0.45 µm filter and the supernatant loaded onto a 5 ml Ni^2+^ column (HisTrap HP, GE Healthcare) in His buffer A. Following extensive washing of the column (>10 column volumes) in His buffer A, the protein was eluted using an increasing gradient of His buffer B (50 mM phosphate buffer pH 8.0, 300 mM NaCl, 500 mM imidazole). Elution fractions were pooled and treated with tobacco etch virus protease (100 µL of 3.3 mg/mL) overnight at 4°C to remove the affinity tag. The cleaved protein was further purified by size exclusion chromatography (Superdex 200 column, GE healthcare) in GST buffer containing 50 mM Tris, pH 8.0, and 125 mM NaCl). The fractions containing protein were pooled and concentrated to 27 mg/ml using an Amicon ultrafiltration device (Millipore). The purity of the protein was assessed by SDS-PAGE and stored at −80°C. Crystallisation screening was undertaking using the hanging-drop vapour-diffusion method and commercially available screens (Hampton Crystal Screen, PEG/Ion, Crystal Screen 2 and PEG/Ion 2). The drops contained 1.5 µl of the protein, to which an equal volume of reservoir solution was mixed, and suspended over 300 µl of reservoir solution at 296 K. Plate shaped diffraction quality crystals were obtained in 1 M sodium acetate trihydrate, 100 mM HEPES pH 7.5, and 50 mM cadmium sulphate hydrate.

### Data collection, structure determination and refinement

Crystals were flash-cooled at 100 K in liquid nitrogen with reservoir solution containing 30% glycerol as a cryoprotectant. Diffraction data were collected from a single crystal at the MX2 crystallography beamline at the Australian Synchrotron. Data were indexed and integrated using iMOSFLM [15] and scaled in AIMLESS [16]. Molecular replacement was undertaken using Phaser [17] and chain A of PDB 2JLM (48% sequence identity) as a search model. Model building and refinement was performed in Coot [18] and Phenix respectively [19].

## Results and Discussion

### Protein production and structure determination

**Figure 1:**
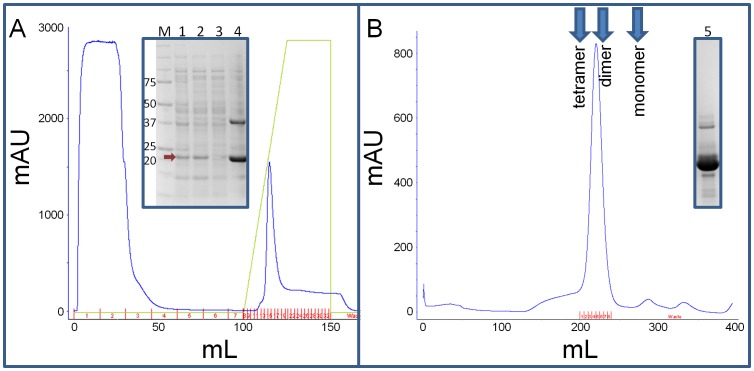
Protein purification profile of *Sa*GNAT. (A) FPLC profile of the affinity purification, with SDS-PAGE insert showing lane 1 - whole bacterial cell lysate; lane 2 - soluble protein fraction of the bacterial cell lysate; lane 3 – flow-through from the affinity column; lane 4 – affinity elution. (B) Size exclusion purification indicating the theoretical elution volumes for monomer, dimer, and trimer, and an SDS PAGE insert lane 5 – showing the final purity of the protein.

To determine the x-ray crystallographic structure of *Sa*GNAT, the gene encoding the protein was cloned into bacterial expression vector pMCSG21 [20] and recombinantly expressed as a 6-His tagged fusion protein in *E. coli* BL21 (DE3) pLysS. The protein was solubly over-expressed using the auto-induction method [14] (see Fig. 1 lanes 1 and 2), and a two-step purification incorporating affinity and size exclusion chromatography resulted in greater than 95% purity (Fig. 1). *Sa*GNAT protein crystals produced in 1 M sodium acetate trihydrate, 100 mM HEPES pH 7.5, and 50 mM cadmium sulphate diffracted to 2.15 Å and were indexed and integrated in the space group *C*2, with unit cell parameters a = 97.5 Å, b = 78.9 Å, c = 66.0 Å, α = 90°, β = 112.0°, γ = 90°. Molecular replacement using Phaser [17] and chain A of PDB model 2JLM was used to place 2 molecules in the asymmetric unit, corresponding to a Matthews coefficient of V_M_ 3.18 Å^3^ Da^−1^ and 61.4% solvent content [21]. Extensive model building and refinement using COOT [18] and Phenix [19] respectively produced a final model with an R_cryst_ and R_free_ 0.18 and 0.22 respectively. All amino acid residues were modelled with the exception of the final C-terminal residue. Coordinate and structure factors have been validated and deposited to Protein Data Bank and assigned the PDB ID code 4MBU. Data-collection and refinement statistics are summarized in Table 1.

**Table 1:** Data collection and refinement statistics.

Wavelength (Å)	0.9537
Resolution range (Å)	33.15 - 2.15 (2.23 - 2.15)
Space group	*C*2
Unit cell (Å)	97.49, 78.86, 66.01, 90, 111.95, 90
Unique reflections	25154 (2479)
Multiplicity	13.4 (13.0)
Completeness (%)	99.38 (98.72)
Mean I/sigma(I)	10.8 (6.0)
Wilson B-factor	17.67
R-merge	0.07 (0.12)
R-work	0.1854 (0.1925)
R-free	0.2226 (0.2648)
RMSD (bonds) (Å)	0.007
RMSD (angles)(°)	1.02
Ramachandran favored (%)	99
Ramachandran outliers (%)	0
Clashscore	2.65
Average B-factor (Å^2^)	21.60
Macromolecules (Å^2^)	20.80
Ligands (Å^2^)	22.80
Solvent (Å^2^)	30.40

Statistics for the highest-resolution shell are shown in parentheses.

### Structure of SaGNAT15

**Figure 2:**
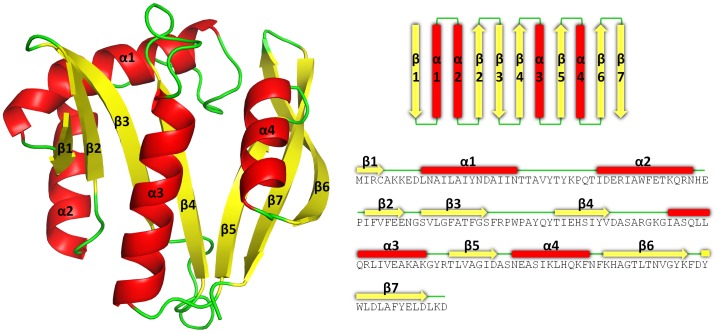
Tertiary structure of SaGNAT in cartoon format, with α-helices, β-strands, and loops colored in red, yellow, and green respectively.

The refined x-ray crystallographic structure revealed *Sa*GNAT to be an α/β protein comprised of 4 α-helices and 7 β-strands, with a topology β1-α1-α2-β2-β3-β4-α3-β5-α4-β6-β7 (Fig. 2). All β-strands are arranged sequentially according to sequence, with the exception of β7, located between strands β5–6. Two central antiparallel β-sheets (β1–4 and β5–8) are splayed between β4 and β5 to create a V-shape in the protein (Fig. 2). The two β-sheets are held together at the V joint by hydrogen bonding located on the N-terminal residues in strands β4–β5, and diverge at Ser^83^ and Ala^117^. This signature feature of GNATs is stabilised by hydrogen bond interactions between water molecules and the amide N and carbonyl O atoms from the protein main chain. The N-terminal arm of the protein is comprised of an antiparallel β-sheet (β1–4) flanked by 3 α-helices (α1, α2 on one side, α3 on the other), and the C-terminal arm is comprised of an antiparallel sheet (β5–7) flanked by α4 on the same side as α3.

**Figure 3:**
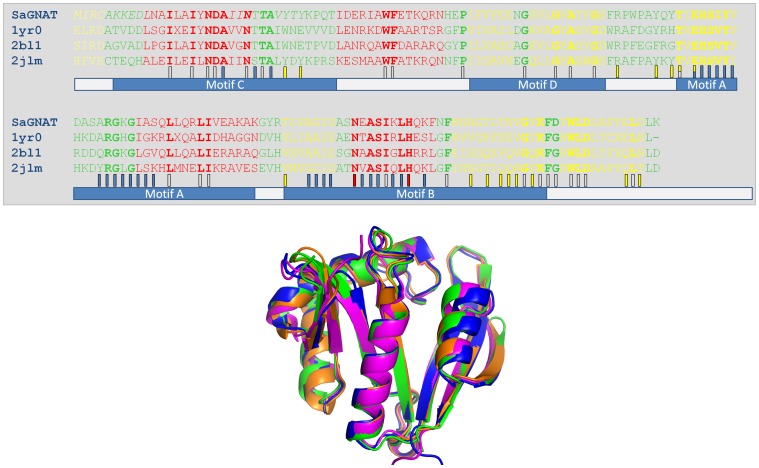
Structure based alignment of the *Sa*GNAT (green) with 3 acyl-transferases and RMSD less than 1 Å. 1YR0 (orange), 1BL1 (blue), and 2JLM (magenta) are crystal structure of phosphinothricin acetyltransferase from *Agrobacterium tumefaciens*, *Pseudomonas aeruginosa*, and *Acinetobacter baylyi* respectively. Blue and red boxes depict conserved CoA binding and active site residues respectively, yellow box indicate residues involved in dimer formation, and unfilled boxes represent strictly conserved residues.

To assess both the sequence and structural similarities of *Sa*GNAT with other GNAT-proteins, BLAST and DALI searches were undertaken. A sequence homology search of the non-redundant database using BLASTP (2.2.28) revealed the most closely related enzyme to be a phosphinothricin N-acetyltransferase (YP_008780792) from *Bacillus cereus*, sharing 60% sequence identity. This low sequence identity between the two closest related homologues is not unusual in the GNAT family, with subfamilies well documented to have highly variable amino-acid sequences, yet retaining very high structural homology [1]. In support of this, a structural homology search using DALI revealed 3 proteins with an rmsd of less than 1 Å, all corresponding to phosphinothricin acetyltransferases (PDB 1yr0/rmsd 0.8/40% sequence identity; PDB 2jlm/rmsd 1.0/48% sequence identity; PDB 2bl1/rmsd 0.9/47% sequence identity). The structural overlay and alignment of these proteins is presented in Fig. 3, with the conserved active site and CoA binding site residues highlighted based on homology with other GNAT family members.

### Quaternary structure of SaGNAT

**Figure 4:**
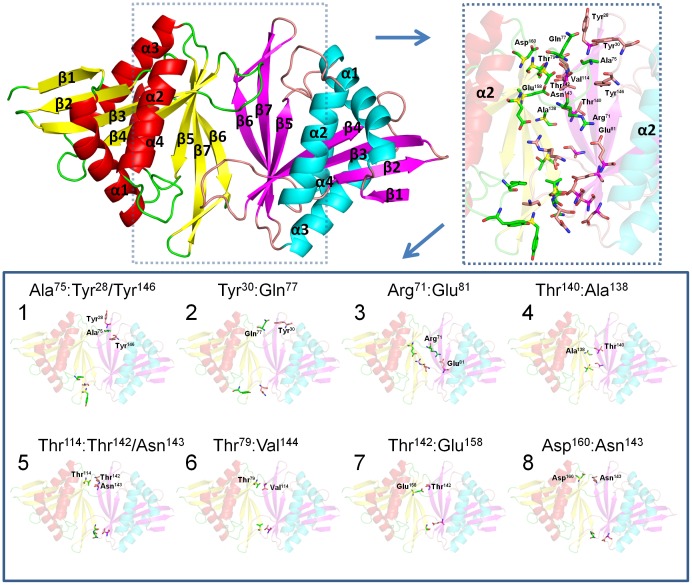
Quaternary structure of SaGNAT showing interacting residues at the dimer interface.

*Sa*GNAT is likely to exist as a dimer based on the crystal structure, structural similarity with homologous proteins, and elution profiles from size exclusion chromatography. In the asymmetric unit of the crystal, two *Sa*GNAT molecules were present with a buried surface area of 1,397 Å^2^, strongly suggesting that this interaction is biologically relevant. Analysis of the inteferaces within the crystal using PISA (Proteins, Interfaces, Structures and Assemblies) also predicted this dimer configuration is likely to represent the biological unit, with other possible crystallographic contacts displaying less than 200 Å^2^ of surface area. Consistent with this result, the structural homology search above confirmed that the proteins with an rmsd of less than 1 Å also exist in the same dimeric configuration. Finally, the elution profile during size exclusion chromatography supports that the protein exists as a dimer in solution (Fig. 1B). The full dimer conformation is presented in Fig. 4, and detailed interactions that mediate the dimer binding are also described. Briefly, the binding interface in comprised Ala75:Tyr28/Tyr146; Tyr30:Gln77; Arg71:Glu81; Thr140:Ala138; Thr114:Thr142/Asn143; Thr79:Val144; Thr142:Glu158; Asp160:Asn143.

### Conclusion

Here, we describe the 2.15 Å structure of a GNAT family member within *S. aureus*. The structure confirms that the protein exhibits the core GNAT fold, and has high structural homology with phosphinothricin acetyltransferases. Consistent with this, the closest homologue identified by BLAST sequence analysis, was also a phosphinothricin acetyltransferase. Putative residues involved in acetyl-CoA and have been identified based on structural homology within the GNAT family.
